# Lactoferrin Is Broadly Active against Yeasts and Highly Synergistic with Amphotericin B

**DOI:** 10.1128/AAC.02284-19

**Published:** 2020-04-21

**Authors:** Kenya E. Fernandes, Kerry Weeks, Dee A. Carter

**Affiliations:** a School of Life and Environmental Sciences, University of Sydney, Sydney, New South Wales, Australia; b NSW Health Pathology, Microbiology Department, Royal North Shore Hospital, Sydney, New South Wales, Australia

**Keywords:** *Candida*, *Cryptococcus*, amphotericin B, antifungal, lactoferrin, yeast

## Abstract

Lactoferrin (LF) is a multifunctional milk protein with antimicrobial activity against a range of pathogens. While numerous studies report that LF is active against fungi, there are considerable differences in the level of antifungal activity and the capacity of LF to interact with other drugs. Here we undertook a comprehensive evaluation of the antifungal spectrum of activity of three defined sources of LF across 22 yeast and 24 mold species and assessed its interactions with six widely used antifungal drugs. LF was broadly and consistently active against all yeast species tested (MICs, 8 to 64 μg/ml), with the extent of activity being strongly affected by iron saturation. LF was synergistic with amphotericin B (AMB) against 19 out of 22 yeast species tested, and synergy was unaffected by iron saturation but was affected by the extent of LF digestion. LF-AMB combination therapy significantly prolonged the survival of Galleria mellonella wax moth larvae infected with Candida albicans or Cryptococcus neoformans and decreased the fungal burden 12- to 25-fold. Evidence that LF directly interacts with the fungal cell surface was seen via scanning electron microscopy, which showed pore formation, hyphal thinning, and major cell collapse in response to LF-AMB synergy. Important virulence mechanisms were disrupted by LF-AMB treatment, which significantly prevented biofilms in C. albicans and C. glabrata, inhibited hyphal development in C. albicans, and reduced cell and capsule size and phenotypic diversity in *Cryptococcus*. Our results demonstrate the potential of LF-AMB as an antifungal treatment that is broadly synergistic against important yeast pathogens, with the synergy being attributed to the presence of one or more LF peptides.

## Abstract (precis)

Lactoferrin (LF) is a multifunctional milk protein with antimicrobial activity against a range of pathogens. While numerous studies report that LF is active against fungi, there are considerable differences in the level of antifungal activity and the capacity of LF to interact with other drugs. Here we undertook a comprehensive evaluation of the antifungal spectrum of activity of three defined sources of LF across 22 yeast and 24 mold species and assessed its interactions with six widely used antifungal drugs.

## INTRODUCTION

Invasive fungal infections are a substantial and growing medical concern due to an increasing population of at-risk immunosuppressed individuals. It is estimated that serious fungal diseases affect more than 150 million people worldwide, causing hundreds of thousands of deaths annually (1). *Aspergillus*, *Candida*, and *Cryptococcus* species are fungal pathogens responsible for a majority of cases of invasive fungal disease (2). Even with the current best antifungal treatment, mortality associated with invasive fungal disease is high, particularly in resource-poor parts of the world. Commonly used antifungal drugs have various limitations, including off-target toxicity, prohibitive expense, unpredictable bioavailability, severe side effects, and the emergence of drug-resistant fungi (3, 4). New compounds with effective antifungal activity are needed, particularly those with broad-spectrum activity and low toxicity. Compounds that can synergistically enhance the effect of current antifungals and lower the necessary therapeutic dose are particularly attractive. As well as greater efficacy, these have the potential to slow the development of resistance by acting on multiple targets (5).

One such synergistic compound is lactoferrin (LF), a multifunctional iron-binding glycoprotein found in various mucosal secretions but present at highest concentrations in milk (6). LF plays a significant physiological role in iron regulation and the innate immune response, and numerous studies have shown LF to have activity against various fungal pathogens, including yeasts and molds (7–10). However, substantial differences in LF activity, ranging from moderate activity to no activity at all, against the same or closely related fungal species have been reported (11). Similarly, studies combining LF with various antifungal drugs, including amphotericin B (AMB), several azoles, and 5-fluorocytosine (5FC) have reported interactions that range from indifferent to synergistic, even with the same combination (7, 12–16). The antifungal effect of LF has typically been attributed primarily to its ability to bind and sequester iron; however, more recent studies suggest a direct interaction with the cell surface and apoptosis-like processes (17–21). LF is also a source of various antimicrobial peptides that are generated upon digestion, with many being reported to contribute to its function and to have antimicrobial activity exceeding that of the native protein (22).

LF is therefore a promising antifungal candidate; however, due to divergent and sometimes conflicting results, work is needed to clarify its true spectrum and degree of activity, possible antifungal mechanisms, and potential for synergistic interactions. We undertook a comprehensive study of the antifungal spectrum of activity of three defined sources of LF. We examined how variations in the composition and purity of LF affect its activity and investigated the interactions of LF with commonly used antifungal drugs. We show that LF has broad and consistent activity against yeasts and that variations among samples greatly influence antifungal activity. In combination, the activity of LF plus AMB was highly synergistic, including *in vivo*, and LF in combination with AMB could effectively suppress certain pathogenic mechanisms.

## RESULTS

### LF samples from different sources vary significantly in purity and composition.

**FIG 1:**
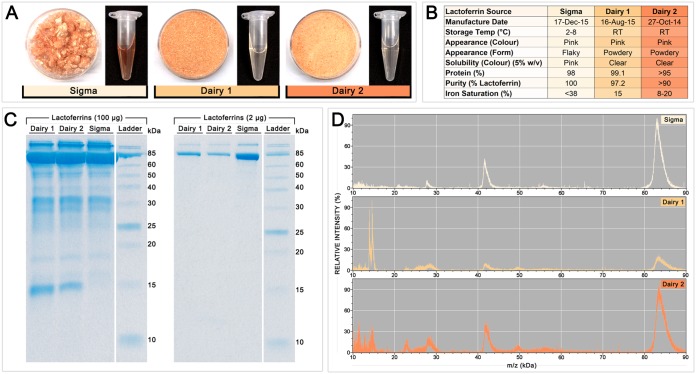
Characteristics of the three LF samples used in this study. (A) Appearance of each LF sample in solid form (left) and dissolved in water at 5% (wt/vol) (right). (B) Summary of the properties of each LF sample, based on visual inspection and manufacturer specifications. RT, room temperature. (C) Protein profiles of the LF samples on SDS-PAGE gels loaded with 100 μg (left) or 2 μg (right). (D) MALDI-TOF mass spectra of each LF sample.

LF samples from three manufacturers were compared to determine if these varied in antifungal activity. The samples differed in appearance and other properties: in solid form, LF from Sigma-Aldrich (LF-S) was flaky, while the LFs from dairies (LF from dairy 1 [LF-D1] and LF from dairy 2 [LF-D2]) were powdery, and in solution at 5% (wt/vol), LF-S was more red in color than LF-D1 and LF-D2, indicating a higher iron saturation (Fig. 1A). Figure 1B summarizes the properties of the different LF samples, based on manufacturer specifications and visual inspection. Notably, LF-S was characterized to be 100% pure, while LF-D1 was 97.2% pure and LF-D2 was >90% pure, and while exact iron saturation was not specified, the possible range given for LF-S was higher at <38%, whereas it was 15% for LF-D1 and 8 to 20% for LF-D2.

The results of an analysis of the composition of the LF samples by SDS-PAGE with high (100 μg) and standard (2 μg) loadings are shown in Fig. 1C (left and right, respectively), and matrix-assisted laser desorption ionization–time of flight (MALDI-TOF) mass spectra are presented in Fig. 1D. Each sample had a major protein band and mass spectrum peak at ∼85 kDa, corresponding to the whole LF protein. A peak at ∼42 kDa in the mass spectrum represents an isotope of whole LF. While smaller bands representing digestion products were apparent in each sample in the 100-μg SDS-PAGE gel, the ∼85-kDa band dominated the LF-S sample, indicating that it was less digested and contained a higher proportion of whole LF than LF-D1 or LF-D2. The mass spectra showed minor peptides, particularly between 20 and 30 kDa and from 50 to 60 kDa, with more being present in the dairy samples. LF-D1 and LF-D2 had peaks at ∼15 kDa in the mass spectra and corresponding ∼15-kDa bands in the 100-μg SDS-PAGE gel that were not present for LF-S.

### LF is broadly active against a diverse range of yeasts.

**FIG 2:**
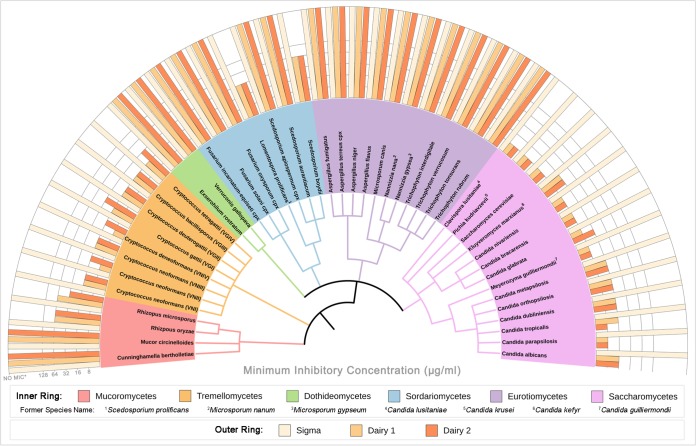
Spectrum of activity of three sources of LF against fungal pathogens. The MIC of each LF sample for a representative strain of 22 yeast and 26 mold species is shown. The middle and inner rings are color coded by class, with the inner ring being a phylogenetic tree generated from reference sequences for each species. NO MIC* indicates that the MIC is above the highest concentration of LF tested (>256 μg/ml for yeasts and >1,024 μg/ml for molds).

To investigate the spectrum of activity of LF against human fungal pathogens and to compare the activity of LF among the different LF samples, MICs for a diverse range of yeast and mold species were determined using CLSI standardized methods. These included predominantly clinical isolates, with some veterinary and environmental isolates along with reference strains being included. Figure 2 shows the LF MIC results for one typical strain from each species, which are grouped phylogenetically by class. For the 22 yeast species tested, the MICs for six commonly used antifungal drugs (AMB, nystatin [NYS], fluconazole [FLC], itraconazole [ITC], voriconazole [VRC], and 5FC) are given in Table 1, and full data for all 100 fungal strains are provided in Data Set S1 in the supplemental material.

**TABLE 1:** LF and antifungal MICs for yeast strains used in this study^*a*^

Species	Strain	MIC (μg/ml)								
		Lactoferrin			Antifungal drugs^*b*^					
		LF-S	LF-D1	LF-D2	AMB	NYS	FLC	ITC	VRC	5FC
*Saccharomycetes*										
Clavispora lusitaniae	M2002	>256	8	8	0.5	8	0.25	0.063	0.004	0.063
	M5619		16	16						
	AMMRL1865		8	8						
	AMMRL1867		16	16						
Pichia kudriavzevii	ATCC 6248	>256	8	8	0.5	8	16	0.25	0.25	8
	AMMRL1878		8	16						
	M7603		8	8						
	M94513		8	8						
Saccharomyces cerevisiae	S288c	>256	8	8	0.25	8	8	0.5	0.25	0.5
	M93149		8	8						
Kluyveromyces marxianus	M1896	>256	16	16	1	8	0.063	0.063	0.004	4
	M230550		16	16						
	AMMRL1826		16	16						
	AMMRL1816		16	16						
Candida nivariensis	AMMRL1900	>256	8	8	0.25	4	4	0.25	0.25	1
Candida bracarensis	AMMRL1891	>256	8	8	0.25	4	2	0.125	0.125	0.5
Candida glabrata	CBS138	>256	8	8	0.25	4	4	0.063	0.125	0.125
	M226760		8	16						
	M221133		8	16						
	M6477		8	8						
Meyerozyma guilliermondii	AMMRL1830	>256	8	8	0.25	4	4	0.125	0.125	0.063
	AMMRL1832		8	8						
	AMMRL1866		8	8						
Candida metapsilosis	M1879	>256	16	16	0.25	4	4	0.125	0.125	0.25
Candida orthopsilosis	M96926	>256	16	16	0.25	4	1	0.125	0.031	0.063
	M230815		16	16						
	M1892		16	16						
	M99271		16	16						
Candida dubliniensis	AMMRL1881	>256	16	16	0.5	8	0.25	0.063	0.004	0.063
	03-058-3327		8	8						
	M230642		16	16						
	AMMRL1792		16	16						
Candida tropicalis	M230640	>256	16	8	1	8	0.5	0.125	0.031	0.25
	M481		16	16						
	M220708		16	16						
	M4754		32	32						
Candida parapsilosis	ATCC 22018	>256	8	8	0.5	8	2	0.125	0.125	0.5
	M7486		16	16						
	M6865		16	16						
	AMMRL1875		16	16						
Candida albicans	SC5314	>256	16	16	0.25	4	1	0.063	0.031	0.125
	03-266-3110		16	32						
	04-022-3139		16	16						
	WM229		16	16						
Geometric mean		NA	12.05	12.63	0.37	5.66	1.49	0.12	0.05	0.31
*Tremellomycetes*										
Cryptococcus neoformans (VNI)	H99/WM148	>256	16	16	0.25	8	2	0.125	0.063	4
	571 148		32	32						
	571 117		16	32						
	CH40-01/WM385		16	32						
Cryptococcus neoformans (VNII)	WM556/RJ-64	>256	8	16	0.25	8	0.25	0.015	0.015	2
	571 114		16	16						
	WM626		16	16						
	C3-1		8	16						
Cryptococcus neoformans (VNIII)	WM628	>256	16	16	0.25	8	1	0.031	0.031	1
	WM01-59		8	16						
Cryptococcus deneoformans (VNIV)	JEC20	>256	8	16	0.25	8	0.5	0.008	0.015	2
	JEC21		32	64						
	WM629		8	16						
	B31-2D		8	16						
Cryptococcus gattii (VGI)	2005/215	>256	16	32	0.25	8	4	0.5	0.25	1
	ENV316		8	32						
	PNG14		8	32						
	V15/571_103		32	16						
Cryptococcus deuterogattii (VGII)	R265	>256	16	16	0.5	8	32	0.5	0.5	2
	97/170		16	16						
	CBS1930		16	32						
	ICB184		16	16						
Cryptococcus bacillisporus (VGIII)	WM161	>256	16	16	0.5	8	1	0.063	0.031	8
	VBP62270		8	16						
	97/427		16	16						
	B13C		32	16						
Cryptococcus tetragattii (VGIV)	MMRL3013	>256	16	32	0.25	8	1	0.125	0.015	2
	M250		16	16						
	WM779		16	16						
	MMRL2650		16	16						
Geometric mean		NA	14.25	20.16	0.30	8.00	1.41	0.07	0.04	2.18

a MICs for LF-D1 and LF-D2 were determined for every strain, while MICs for LF-S and antifungal drugs (AMB, NYS, FLC, ITC, VRC, and 5FC) were determined for a single strain from each species.

b AMB, amphotericin B; NYS, nystatin; FLC, fluconazole; ITC, itraconazole; VRC, voriconazole; 5FC, 5-fluorocytosine; NA, not applicable.

An MIC could not be determined for LF-S within the tested range for any yeast (>256 μg/ml) or mold (>1,024 μg/ml) strain. The dairy LFs, however, were effective against every yeast strain tested, including *Cryptococcus*, *Candida*, *Clavispora*, *Pichia*, *Saccharomyces*, *Kluyveromyces*, and *Meyerozyma* spp., with MICs ranging from 8 to 64 μg/ml. LF-D1 had significantly lower MICs than LF-D2 (*P* = 0.0101) for the *Tremellomycetes*, but there was no significant difference (*P* = 0.3445) for the *Saccharomycetes*. For yeast strains for which the MICs of other antifungal drugs were determined, the dairy LFs were found to be equally effective regardless of susceptibility to any other drug. The ranges of MICs were as follows: AMB, 0.25 to 1 μg/ml; NYS, 4 to 8 μg/ml; FLC, <0.125 to 32 μg/ml; ITC, <0.016 to 1 μg/ml; VRC, <0.008 to 1 μg/ml; and 5FC, <0.063 to 8. Four of the mold species had MICs with the dairy LFs: Rhizopus oryzae (64 μg/ml), Rhizopus microsporum (32 to 64 μg/ml), Lomentospora prolificans (32 μg/ml), and Scedosporium boydii (64 μg/ml). All other species (*Cunninghamella*, *Mucor*, *Exserohilum*, *Verruconis*, *Fusarium*, *Scedosporium*, *Aspergillus*, *Microsporum*, *Nannizzia*, and *Trichophyton*) had no MIC within the tested range (>1,024 μg/ml). As it was the most effective, LF-D1 was used for all further tests. Yeasts rather than molds were further investigated, as they were more consistently and broadly susceptible to LF.

### LF synergizes with AMB but not with other antifungal drugs.

**FIG 3:**
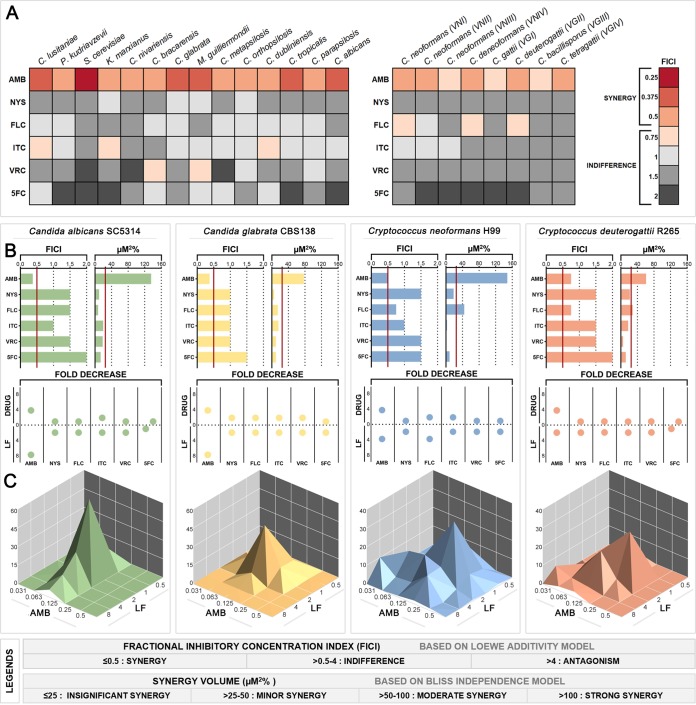
Interaction of LF with various antifungal drugs against yeasts. (A) Heatmap showing the FICI values for LF-D1 combined with each of six antifungal drugs against 22 yeast species, based on abbreviated checkerboards. (B) Summary of interactions between LF-D1 and antifungal drugs against yeast reference strains C. albicans SC5314, C. glabrata CBS138, C. neoformans H99, and C. deuterogattii R265 based on full checkerboards. (Left) FICI based on the Loewe additivity model, with values for synergy being indicated to the left of the red cutoff line; (right) micromolar squared percent based on the Bliss independence model and calculated by MacSynergy II, with values for synergy being indicated to the right of the red cutoff line; (bottom) fold decrease in the concentration of each agent required to inhibit growth when it was used in combination compared to that when it was used alone. (C) Three-dimensional dose-response surfaces generated in MacSynergy II from full checkerboard data showing significant synergy volumes, represented as peaks above the flat plane, for the combination of LF-AMB in the same four fungal species. The legends at the bottom show the cutoff values for interactions for each model.

To determine how LF interacts with other antifungal agents and find synergistic parings, LF-D1 was tested in combination with commonly used antifungal drugs (AMB, NYS, FLC, ITC, VRC, and 5FC) for activity against 22 yeast species using an abbreviated diagonal-sampling checkerboard methodology (23). The resulting fractional inhibitory concentration indexes (FICIs) for each combination are shown in Fig. 3A, with individual fractional inhibitory concentrations (FICs) and fold changes for the LF-AMB combinations being listed in Table 2. Full data for all combinations are given in Data Set S2. The combination of LF and AMB was synergistic (FICI = 0.25 to 0.5) against all 14 *Saccharomycetes* species, with the lowest FICI being for Saccharomyces cerevisiae (FICI = 0.25). LF-AMB was synergistic (FICI = 0.5) against five of the eight *Cryptococcus* genotypes/species tested, with an indifferent interaction being shown against the remaining three genotypes/species (FICI = 0.75). The activities of the remaining combinations of LF plus NYS, LF plus FLC, LF plus ITC, LF plus VRC, and LF plus 5FC were indifferent against every strain. The FICI calculation requires at least a 4-fold reduction in the MIC of both agents to produce an FICI of ≤0.5; however, in the case of antifungals and LF, reducing the MIC of the drug is more critical than reducing the MIC of LF. When examining this, a 4-fold decrease for ITC was seen for Candida lusitaniae and Kluyveromyces marxianus and a 4-fold decrease for VRC was seen for Candida bracarensis and Meyerozyma guilliermondii (Data Set S2). No antagonism was observed with any combination.

**TABLE 2:** FICI and fold change data for the combination of LF and AMB in yeast species^*a*^

Species	Strain	Amphotericin B				Lactoferrin (dairy 1)				FICI^*b*^
		MIC*_X_* (μg/ml)	MIC*_Y_* (μg/ml)	FIC_A_ (μg/ml)	Fold change	MIC*_X_* (μg/ml)	MIC*_Y_* (μg/ml)	FIC_B_ (μg/ml)	Fold change	
*Saccharomycetes*										
Clavispora lusitaniae	M2002	0.5	0.125	0.25	4	8	1	0.125	8	**0.375**
Pichia kudriavzevii	ATCC 6248	0.5	0.125	0.25	4	8	2	0.25	4	**0.5**
Saccharomyces cerevisiae	S288c	0.25	0.031	0.125	8	8	1	0.125	8	**0.25**
Kluyveromyces marxianus	M1896	1	0.25	0.25	4	16	4	0.25	4	**0.5**
Candida nivariensis	AMMRL1900	0.25	0.063	0.25	4	8	2	0.25	4	**0.5**
Candida bracarensis	AMMRL1891	0.25	0.063	0.25	4	8	2	0.25	4	**0.5**
Candida glabrata	CBS138	0.25	0.063	0.25	4	8	1	0.125	8	**0.375**
Meyerozyma guilliermondii	AMMRL1830	0.25	0.063	0.25	4	8	1	0.125	8	**0.375**
Candida metapsilosis	M1879	0.25	0.063	0.25	4	16	4	0.25	4	**0.5**
Candida orthopsilosis	M96926	0.25	0.063	0.25	4	16	4	0.25	4	**0.5**
Candida dubliniensis	AMMRL1881	0.5	0.125	0.25	4	16	4	0.25	4	**0.5**
Candida tropicalis	M230640	1	0.25	0.25	4	16	2	0.125	8	**0.375**
Candida parapsilosis	ATCC22018	0.5	0.125	0.25	4	8	2	0.25	4	**0.5**
Candida albicans	SC5314	0.25	0.063	0.25	4	16	2	0.125	8	**0.375**
*Tremellomycetes*										
Cryptococcus neoformans (VNI)	H99/WM148	0.25	0.063	0.25	4	16	4	0.25	4	**0.5**
Cryptococcus neoformans (VNII)	WM556/RJ-64	0.25	0.063	0.25	4	8	2	0.25	4	**0.5**
Cryptococcus neoformans (VNIII)	WM628	0.25	0.125	0.5	2	16	4	0.25	4	0.75
Cryptococcus deneoformans (VNIV)	JEC20	0.25	0.063	0.25	4	8	2	0.25	4	**0.5**
Cryptococcus gattii (VGI)	2005/215	0.25	0.063	0.25	4	16	8	0.5	2	0.75
Cryptococcus deuterogattii (VGII)	R265	0.5	0.125	0.25	4	16	8	0.5	2	0.75
Cryptococcus bacillisporus (VGIII)	WM161	0.5	0.25	0.5	2	16	4	0.25	4	0.75
Cryptococcus tetragattii (VGIV)	MMRL3013	0.25	0.063	0.25	4	16	4	0.25	4	**0.5**

a MIC*_X_* is the MIC of the agent alone, MIC*_Y_* is the MIC of the agent in combination, each FIC is calculated as (MIC*_X_*/MIC*_Y_*), and FICI is the sum of FIC_drug A_ and FIC_drug B_.

b Values in bold indicate synergistic activity.

The reference strains of four clinically significant yeast species, Candida albicans SC5314, Candida glabrata CBS138, Cryptococcus neoformans H99, and Cryptococcus deuterogattii R265, were chosen for further investigation using full checkerboard assays and two models to assess synergy (Fig. 3B). FICI results (Fig. 3B, left) and the fold decrease (Fig. 3B, bottom) for each agent again found LF-AMB to be the only combination showing synergy. The activity of LF-AMB against C. deuterogattii was indifferent (FICI = 0.75); however, there was a 4-fold decrease in the requirement for AMB.

FICI is based on Loewe additivity, which assumes that both agents have the same mechanism of action, while micromolar squared percent (Fig. 3B, right), calculated by use of the MacSynergy II program, is based on Bliss independence and does not have this assumption. In this model, LF-AMB produced strong synergy against C. albicans (135.48 μM^2^%) and C. neoformans (148.1 μM^2^%) and moderate synergy against C. glabrata (77.46 μM^2^%) and C. deuterogattii (60.76 μM^2^%). In addition, LF-FLC showed minor synergy against C. neoformans (44.27 μM^2^%) and C. deuterogattii (28.77 μM^2^%). Figure 3C shows the dose-response surfaces for LF-AMB generated with MacSynergy II. The dose-response surfaces for all other combinations can be found in Data Set S2. Overall, the combination of LF plus AMB showed strong and consistent synergy across multiple yeast species and strains. Although LF-FLC, LF-ITC, and LF-VRC were synergistic or reduced the amount of drug needed by 4-fold in some instances, the results were strain specific and were not consistently effective, and so these combinations were not pursued further.

### Iron saturation has a major effect on the activity of LF alone but has little or no effect on LF-AMB synergy.

The ability of LF to bind molecules of iron with a very high affinity, making them unavailable to pathogens, has been shown to be an important component of its antimicrobial activity (24). Therefore, to further investigate the effect of iron saturation levels on the activity of the LF samples used in this study, the samples were saturated with iron to produce iron-rich holo-LF or extensively dialyzed to produce iron-deplete apo-LF. The amount of iron in each sample was quantified by inductively coupled plasma mass spectrometry (ICP-MS), and the activity of each sample alone or in combination with AMB was tested in C. albicans SC5314, C. glabrata CBS138, C. neoformans H99, and C. deuterogattii R265 (Table 3). ICP-MS results were consistent with the original data (Fig. 1) showing that LF-S had far more iron (27.7%) than either LF-D1 (14.5%) or LF-D2 (10.3%). Following saturation, high iron levels of 78.3% for holo-LF-S, 72.9% for holo-LF-D1, and 71.4% for holo-LF-D2 were achieved, while following dialysis, these dropped to 5.2% for apo-LF-S, 1.1% for apo-LF-D1, and 2.6% for apo-LF-D2. The activity of the holo-LF-D1 and holo-LF-D2 samples decreased significantly, such that no MIC (>256 μg/ml) could be found within the tested range, while there was no change for holo-LF-S, with no MIC being found for any species.

**TABLE 3:** Effect of iron saturation on antifungal activity of LF and synergy with AMB

Lactoferrin source	Treatment (name)	Iron saturation (%)^*a*^	C. albicans SC5314		C. glabrata CBS138		C. neoformans H99		C. deuterogattii R265	
			MIC (μg/ml)	FICI^*b*^	MIC (μg/ml)	FICI^*b*^	MIC (μg/ml)	FICI^*b*^	MIC (μg/ml)	FICI^*b*^
Sigma	Control (LF-S)	27.7	>256	**0.5**	>256	**0.5**	>256	**0.5**	>256	**0.5**
	Saturated (holo-LF-S)	78.3	>256	**0.5**	>256	**0.5**	>256	**0.5**	>256	**0.5**
	Dialyzed (apo-LF-S)	5.2	256	**0.5**	256	**0.5**	256	**0.5**	128	**0.5**
Dairy 1	Control (LF-D1)	14.5	16	**0.375**	16	**0.5**	16	**0.5**	8	**0.375**
	Saturated (holo-LF-D1)	72.9	>256	**0.375**	>256	**0.5**	>256	**0.5**	>256	**0.375**
	Dialyzed (apo-LF-D1)	1.1	32	**0.375**	32	**0.5**	32	**0.5**	16	**0.375**
Dairy 2	Control (LF-D2)	10.3	16	**0.375**	16	**0.5**	16	**0.5**	8	**0.375**
	Saturated (holo-LF-D2)	71.4	>256	**0.375**	>256	**0.5**	>256	**0.5**	>256	**0.375**
	Dialyzed (apo-LF-D2)	2.6	32	**0.375**	32	**0.5**	32	**0.5**	16	**0.375**

a Iron saturation was determined by ICP-MS.

b FICI of LF from the specified source plus AMB. Values in bold indicate synergistic activity.

The activity levels of apo-LF-D1 and apo-LF-D2 were retained (MIC, 16 to 32 μg/ml) and were slightly higher than those of LF-D1 and LF-D2, most likely due to some dilution occurring during the extensive dialysis process. In addition, apo-LF-S displayed activity against every species (MIC, 128 to 256 μg/ml), whereas LF-S had no activity. The FICIs for all four species were unaffected by the iron saturation level of LF. These results indicate that iron chelation plays a major role in the activity of LF alone but has little or no effect on the synergistic combination of LF and AMB.

### Combination therapy with LF-AMB significantly decreases death and reduces fungal burden in a Galleria mellonella model of infection.

**FIG 4:**
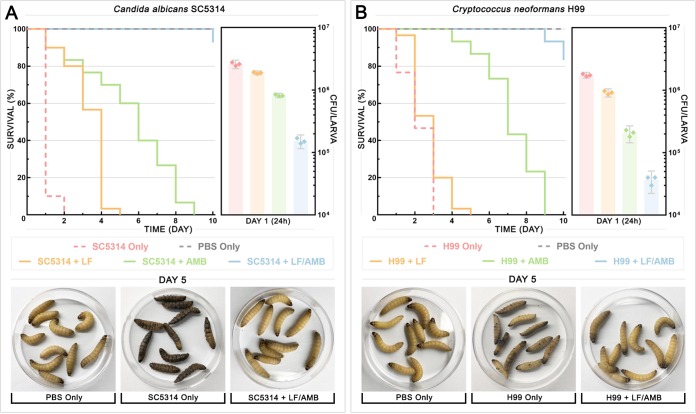
Combination therapy with LF and AMB in Galleria mellonella infection model. G. mellonella larvae were infected with an inoculum of 10^8^ cells of either C. albicans SC5314 (A) or C. neoformans H99 (B), and treatment with LF-D1 (1,024 μg/ml), AMB (128 μg/ml), or both combined was administered. (Left) Survival plots for 3 replicate experiments combined (*n* = 30). For each replicate, a group of 10 larvae for each treatment was monitored for survival over 10 days. (Right) Fungal burden for 3 replicate experiments combined (*n* = 3). For each replicate, three larvae from each group were sacrificed after 24 h, homogenized, and backplated, and the colonies were counted to determine the number of CFU per larva. (Bottom) Photographs of representative larvae at day 5 that had been mock infected (PBS Only), infected and left untreated (SC5314 Only or H99 Only), and infected and treated with combination therapy (SC5314 + LF/AMB or H99 + LF/AMB).

Following confirmation of a synergistic interaction *in vitro*, the *in vivo* efficacy of LF-D1 alone and in combination with AMB against C. albicans SC5314 or C. neoformans H99 was tested in Galleria mellonella (wax moth) larvae. The G. mellonella infection model is an established model host system that has been used to study a number of clinically important fungi, including *Candida* and *Cryptococcus* species, and the results obtained with this model show a strong correlation with the results obtained using mammalian models (25–27). A range of drug concentrations for LF (64 to 1,024 μg/ml) and AMB (8 to 128 μg/ml) was initially tested, with the full data provided in Data Set S3. Survival curves (Fig. 4A and B, left), fungal burdens (Fig. 4A and B, right), and photographs of the larvae (Fig. 4A and B, bottom) are shown for larvae infected with C. albicans (Fig. 4A) and C. neoformans (Fig. 4B) and treated with the most effective tested concentrations of LF (1,024 μg/ml) and AMB (128 μg/ml). After injecting G. mellonella larvae with either strain, they were treated with phosphate-buffered saline (PBS), monotherapy, or combination therapy at 30 min postinoculation. For the untreated control, C. albicans killed the larvae significantly faster than C. neoformans, with 90% and 25% of the larvae being dead after 1 day, respectively. C. albicans also induced melanization far more rapidly and intensely than C. neoformans. LF monotherapy prolonged the survival of 90% of the larvae infected with C. albicans for up to 3 days (*P* < 0.0001) and 20% of the larvae infected with C. neoformans for up to 2 days (*P* = 0.0416). AMB monotherapy was significantly (*P* < 0.0001) more effective than LF monotherapy against both C. albicans and C. neoformans, prolonging the survival of 90% of larvae for up to 7 days and 100% of the larvae for up to 6 days, respectively. However, while survival was prolonged in both monotherapy treatments, all larvae were dead by day 9.

The LF-AMB combination was significantly more effective than monotherapy treatments (*P* < 0.0001), with 28 and 25 out of 30 larvae infected with C. albicans and C. neoformans, respectively, surviving past day 10. Photographs of the larvae at day 5 show the stark difference between dead and melanized untreated control larvae and those treated with LF-AMB combination therapy (Fig. 4). The fungal burden mirrored these results, with the number of CFU per larva after 24 h decreasing significantly after all drug treatments compared to that after no treatment (controls) (*P* < 0.0001) for both species. The combination of LF and AMB reduced the fungal burden more than 5-fold compared to that for AMB alone for both species and by more than 12-fold and 25-fold compared to that for LF alone for C. albicans and C. neoformans, respectively.

### Scanning electron microscopy shows evidence of cell surface interaction following LF and LF-AMB treatments.

**FIG 5:**
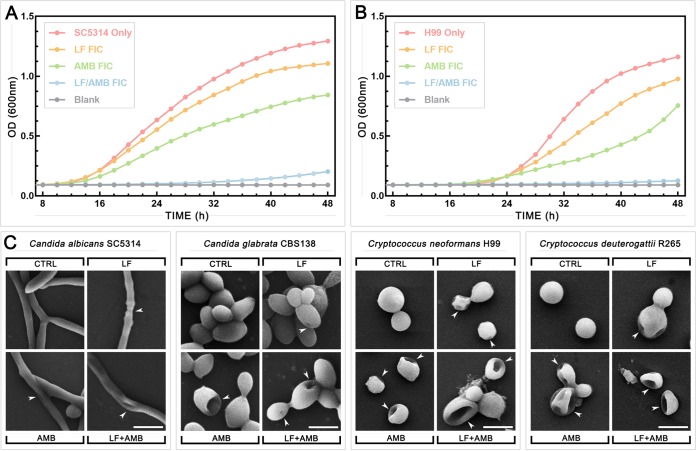
Growth dynamics and morphological changes of *Candida* and *Cryptococcus* grown in the presence of LF, AMB, and LF-AMB. Time-kill curves of C. albicans SC5314 (A) and C. neoformans H99 (B) incubated at 35°C with shaking at 180 rpm and either left untreated or treated with the LF-D1 FIC, AMB FIC, or LF-AMB FIC averaged across three independent replicates. OD, optical density. (C) Scanning electron microscopy images of the four yeast species after 48 h (C. albicans SC5314, C. glabrata CBS138) or 72 h (C. neoformans H99, C. deuterogattii R265) of growth untreated or in the presence of 1/2 MIC of LF-D1 or AMB or 1/2 FIC of LF-AMB (the MICs and FICs for each species can be found in Table 2). Magnifications = ×2,000 for C. albicans, C. neoformans, and C. deuterogattii and ×4,000 for C. glabrata. Bars = 5 μm for C. albicans, C. neoformans, and C. deuterogattii and 2.5 μm for C. glabrata. White arrowheads indicate the morphological changes following treatment.

In addition to iron chelation, a direct interaction of LF with the fungal surface, leading to cell membrane damage, has been reported to be an antifungal mechanism (11). To investigate this, scanning electron microscopy (SEM) was used to visualize morphological changes to treated cells. The effects of the FICs of the drug alone and the drug in combination on growth over time were first compared by the use of time-kill curves for C. albicans SC5314 (Fig. 5A) and C. neoformans H99 (Fig. 5B). LF-D1 and AMB at FICs had a minor effect on growth compared to the growth of the untreated control, while the LF-AMB combination completely suppressed growth to nearly background levels, although there was a slight increase toward the end of the 48-h test period. Based on this, 1/2 MICs and 1/2 FICs were chosen for SEM treatment to enable some growth and reproduction of the cells. Full checkerboard assays were then performed for C. albicans SC5314, C. glabrata CBS138, C. neoformans H99, and C. deuterogattii R265. After 48 and 72 h of incubation for the *Candida* and *Cryptococcus* strains, respectively, cells were taken from the wells corresponding to 1/2 MIC for LF-D1 and AMB and 1/2 FIC for LF-AMB and prepared for SEM (Fig. 5C).

For both C. neoformans and C. deuterogattii, control samples had regular, smooth, round cells. The majority of cells following LF treatment were normal, with a few (approximately 10%) looking shriveled and sunken, suggesting that LF interacts with the surface of the cells in some capacity. For the AMB and LF-AMB treatments, a large proportion (approximately 40%) of the cells were damaged, with the damage ranging from slight to severe collapse. In C. albicans and C. glabrata, most cells were normal in all treatments, with a few (approximately 5%) displaying morphological alterations, suggesting that the subinhibitory concentrations used did not induce the substantial cell damage that had been seen in *Cryptococcus*. With LF treatment in C. albicans, hyphae showed thinning in some areas, and with AMB and LF-AMB treatments, some hyphae appeared to have developed small lesions. In C. glabrata, control cells were smooth ovals present in large clusters. With LF treatment, small pores were present in some cells, and with AMB and LF-AMB treatments, cells formed much smaller clusters, with some cells showing damage ranging from small pores to major collapse that was similar to that seen in the *Cryptococcus* samples.

### LF-AMB acts synergistically to prevent and damage *Candida* biofilms and reduce hyphal growth.

**FIG 6:**
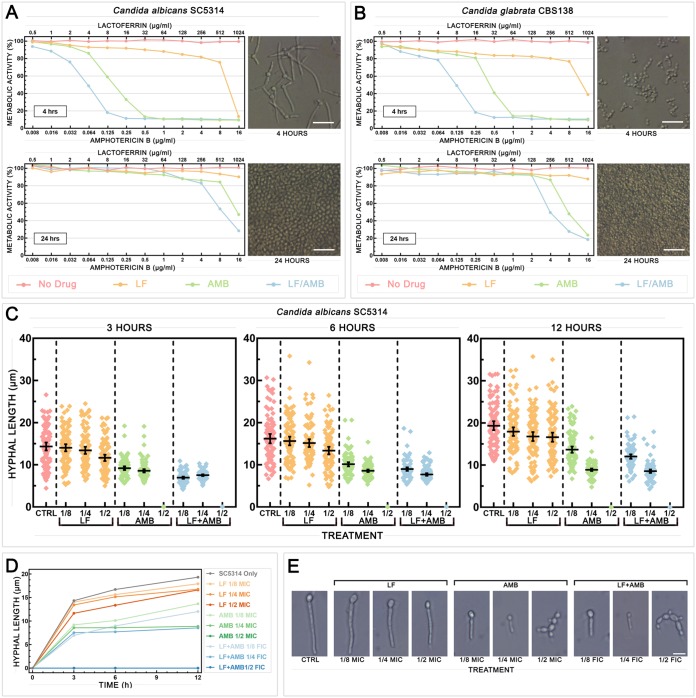
Effect of synergistic treatment with LF and AMB on biofilm and hyphal formation in *Candida*. Effect of subinhibitory concentrations of LF-D1, AMB, or LF-AMB on biofilm formation (4 h) and mature biofilms (24 h) in C. albicans SC5314 (A) and C. glabrata CBS138 (B). (Left) Metabolic activity at different drug concentrations, measured by the XTT reduction assay and averaged across three independent replicates; (right) representative light microscopy images of biofilms before treatment. (C) Hyphal length of C. albicans SC5314 after 3, 6, or 12 h of growth under hypha-inducing conditions (DMEM at 37°C with shaking at 180 rpm) either with no treatment or in the presence of 1/2, 1/4, or 1/8 MIC of LF-D1 or AMB or 1/2, 1/4, or 1/8 FIC of LF-AMB (the MICs and FICs for each species can be found in Table 2) (*n* = 100). Error bars show the mean ± 95% confidence interval. (D) Hyphal growth curve of C. albicans SC5314 with no treatment or in the presence of various drug treatments over time. (E) Representative light microscopy images showing hyphal development following each treatment at 12 h. Bar = 10 μm.

In *Candida* infections, the formation of biofilms is significant, as they are intrinsically resistant to many current antifungal drugs, requiring high-dose therapy that can result in severe side effects (28, 29). The efficacy of LF-AMB against biofilms formed by C. albicans SC5314 and C. glabrata CBS138 was assessed using an 2,3-bis-(2-methoxy-4-nitro-5-sulfophenyl)-2H-tetrazolium-5-carboxanilide salt (XTT) reduction assay to measure metabolic activity (Fig. 6A and B). Cells were allowed to grow for 4 h to investigate biofilm formation (Fig. 6A and B, top) or 24 h to investigate mature biofilms (Fig. 6A and B, bottom), before nonadherent planktonic cells were removed and treatments were applied. At 4 h, synergistic 80% FICI (FICI_80_) values were seen for LF-AMB (0.258 with 8-μg/ml LF plus 0.125-μg/ml AMB for C. albicans and <0.258 with 16-μg/ml LF plus 0.25-μg/ml AMB for C. glabrata), reducing the amount of drug required 4-fold for AMB and at least 128-fold for LF. At 24 h, an FICI_80_ for LF-AMB was not achieved for C. albicans within the tested range; however, the combination, which had an AMB concentration 2-fold lower than that of AMB alone, produced inhibition comparable to that achieved with AMB alone. The FICI_80_ for C. glabrata could not be calculated, as there were no LF MIC_80_ values, but inhibition was achieved with 1,024-μg/ml LF plus 16-μg/ml AMB, which, again, was a concentration 2-fold lower than that of AMB alone. These results indicate that the combination of LF and AMB is highly effective at preventing biofilm formation, reducing the amounts of both drugs required. While it was not as potent against mature biofilms, the combination of LF and AMB was still more effective than either drug alone.

To further investigate the effect of drug treatment on hyphal development, C. albicans SC5314 cells were grown under hypha-inducing conditions (Dulbecco’s modified Eagle medium [DMEM] at 37°C with shaking at 180 rpm) with 1/2, 1/4, and 1/8 MIC or the FIC of the drug added. Figure 6C shows the hyphal length measured at 3, 6, and 12 h for each treatment, with the average value for each treatment at each time point being plotted in Fig. 6D and with representative photographs being shown in Fig. 6E. Treatment with 1/8 or 1/4 FIC of LF-AMB substantially reduced the hyphal length at every time point (*P* = 0.0001), while 1/2 FIC of LF-AMB completely prevented hyphal formation, resulting instead in chains of connected yeast cells.

### LF-AMB acts synergistically to reduce morphological changes associated with virulence in *Cryptococcus*.

**FIG 7:**
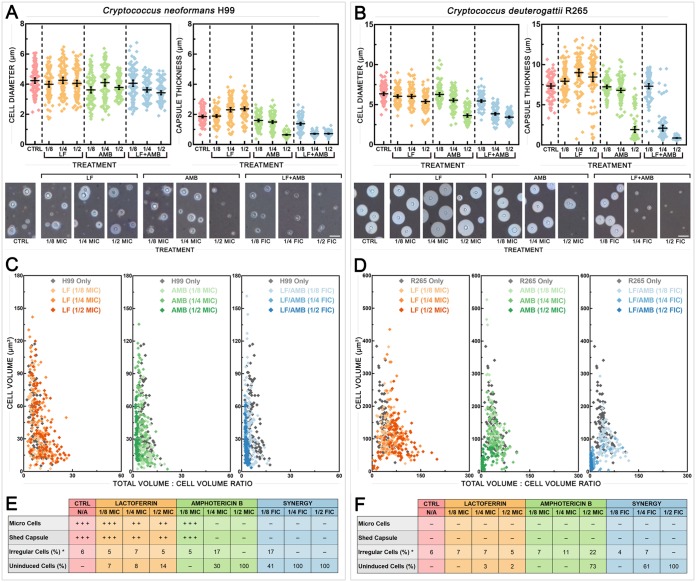
Effect of synergistic treatment with LF and AMB on the induction of morphological variants in *Cryptococcus*. (A and B) Cell diameter (left) and capsule thickness (right) of C. neoformans H99 (A) and C. deuterogattii R265 (B) grown under capsule-inducing conditions (DMEM with 5% CO_2_ at 37°C for 5 days) either with no treatment or in the presence of 1/2, 1/4, or 1/8 MIC of LF-D1 or AMB or 1/2, 1/4, or 1/8 FIC of LF-AMB (the MICs and FICs for each species can be found in Table 2) (*n* = 100). Error bars show the mean ± 95% confidence interval. (Bottom) Indian ink-stained preparations showing the variation in capsule and cell size for each treatment. Bars = 10 μm for C. neoformans and 20 μm for C. deuterogattii. (C and D) To demonstrate the relationship between capsule production and cell size, the average cell volume is plotted against the average total volume (cell plus capsule) for 100 individual cells under each treatment for C. neoformans H99 (C) and C. deuterogattii R265 (D). Cells for which the results sit farther along the *x* axis possess a larger relative capsular volume, while cells for which the results sit farther along the *y* axis possess larger cell volumes. (E and F) The presence and frequency of morphological variants in C. neoformans H99 (E) and C. deuterogattii R265 (F) following the various treatments. *, irregular cell numbers are expressed as a percentage of the number of induced cells; N/A, not applicable.

In *Cryptococcus*, morphological changes associated with virulence include cell and capsule enlargement, the release of shed capsule, and the production of variant populations of cells known as giant cells (≥15 μm) and micro cells (≤1 μm) (30). To investigate the effect of LF-D1 alone and in combination with AMB on these phenotypes, C. neoformans H99 (Fig. 7A) and C. deuterogattii R265 (Fig. 7B) cells were grown under capsule induction conditions (DMEM with 5% CO_2_ at 37°C for 5 days) with drugs present at 1/2, 1/4, and 1/8 MIC or FIC (of LF-AMB). The induction conditions were designed to simulate *in vivo* conditions and are known to induce phenotypic changes associated with virulence (31, 32).

Cell diameter (Fig. 7A and B, left) and capsule thickness (Fig. 7A and B, right) were measured, with representative photographs also being shown (Fig. 7A and B, bottom). The *P* values for all following treatments were <0.01. For C. neoformans H99, treatment with 1/4 or 1/2 FIC of LF-AMB significantly decreased the cell size compared to that for the control, and treatment with all three concentrations of LF-AMB significantly decreased the capsule thickness. The effect of LF-AMB on the capsule was significantly more pronounced than the corresponding AMB treatment at 1/8 and 1/4 FIC, but there was no difference at 1/2 MIC or FIC. For C. deuterogattii, treatment with all three concentrations of LF-AMB significantly decreased the cell size compared to that for the control, and LF-AMB produced a significantly greater effect at 1/4 FIC than the corresponding AMB treatment. Similar to the findings for C. neoformans, treatment with 1/4 or 1/2 FIC of LF-AMB significantly decreased the capsule thickness. LF-AMB induced significantly less capsule at 1/4 and 1/2 FIC than the corresponding AMB treatments. Overall, the combination of LF-AMB resulted in a reduced cell size and a reduced capsule size in both C. neoformans and C. deuterogattii to an extent similar to or greater than that of AMB treatment, resulting in cells that appeared to be effectively uninduced.

The relationship between capsule production and cell size in individual cells is further illustrated in Fig. 7C and D, where the *x* axis shows the relative volume of capsule for each cell independently of the cell volume, which was standardized to 1, while the *y* axis shows the corresponding cell volume. In both species, LF treatments produced the largest range in capsule thickness and cell volume, exceeding those for the control. The AMB and LF-AMB treatments caused different responses in the two species: for C. neoformans, the results for the cells clustered along the *y* axis with increasing drug levels, indicating a smaller relative capsule and larger cellular volumes, while for C. deuterogattii, the results for the cells clustered close to the origin, indicating a smaller capsule and lower cellular volumes.

Morphological variants were present in some of the cultures and were affected by the presence of drugs (Fig. 7A and B). The data are summarized in Fig. 7E and F, where micro cells were cells ≤1 μm, shed capsule was blebs of capsule released into the medium, irregular cells were abnormal elongated cells, and uninduced cells were cells that did not appear to increase in cell or capsule size compared to the sizes for cells grown under standard noninducing conditions (31). Micro cells and shed capsule were produced only in C. neoformans and were completely prevented by LF-AMB at every concentration tested. Both C. neoformans and C. deuterogattii control cultures had similar numbers of irregular cells, and no cells were uninduced. Increasing concentrations of LF-AMB increased both the percentage of uninduced cells and the proportion of induced cells that were irregular.

## DISCUSSION

### Iron saturation and level of digestion vary significantly among LF sources and strongly affect antifungal activity and drug synergy.

The current study set out to characterize and test the activities of LFs from three separate sources against a large suite of clinically important yeast and mold species, with the aim of providing a comprehensive analysis of antifungal activity and determining factors that might influence the lack of consistency seen across different published studies. In doing this, we were able to show that the activity of LF is very consistent across yeast species but that the extent of the activity is strongly affected by the properties of the LF sample. While other studies have reported a wide range of activities for LF in the same or similar species (e.g., MICs ranging from 200 to >6,400 μg/ml for C. albicans across various studies) and large species-specific differences (11), we found the MICs across a variety of yeast species to be within a similar range when using the same LF sample but to vary significantly with different LF samples.

The purest LF sample, LF-S, purchased from Sigma-Aldrich, was the least antifungal (Fig. 2; Table 1). LF-S had a higher level of iron saturation and substantially fewer digestion products than the more active dairy LFs (Fig. 1). Iron chelation is well documented to be an antifungal mechanism of LF, and several other iron chelators have also been found to have antifungal activity over a range of concentrations (7). Studies have shown that the addition of iron during LF treatment completely reverses growth inhibition in yeasts (7, 17, 33); however, iron-independent mechanisms are also believed be involved (11). While we found that the initial differences in iron saturation of each LF sample aligned with their different activities and that the saturation of the dairy LFs with iron caused them to lose their activity, the stripping of iron from LF-S produced only a modest result, and it remained substantially less active than the iron-depleted dairy LF samples (Table 3). This confirmed a role of iron chelation in the antifungal activity of LF in yeasts and demonstrated that this is not the only mechanism of action.

Proteolytic enzymes and acid digestion cleave LF into a range of smaller peptides (23). The two dairy LF samples had a substantially greater number and diversity of peptides than the LF-S sample, but the number and diversity of peptides were similar to those in a pure commercial LF preparation supplied by MP Biochemicals (7) (Fig. 1C and D). Although reports of the antifungal activities of some LF peptides vary widely (e.g., in C. albicans, the 3-kDa peptide lactoferricin has MICs ranging from 0.8 to 400 μg/ml), these are generally considered to have substantially more antifungal activity than LF itself, and their presence could significantly affect the MIC (11). In addition to the peptides present in the initial sample, it is quite likely that further peptides are produced during antifungal testing by the action of secreted fungal enzymes. Holo-LF is known to be more resistant to digestion than apo-LF; thus, iron saturation is likely to affect peptide composition and may be another factor contributing to the lack of antifungal activity seen in LF-S (34).

Our results indicate that the lack of consistency across previous studies can be explained by variations in LF sources and methodologies. Many past studies have used a single source of LF, and there is often no evidence of characterization beyond the manufacturer’s specifications (7, 13, 15, 33, 35), so that iron saturation levels and peptide composition, which we have shown significantly affect antifungal activity, are unknown. Furthermore, different antifungal susceptibility testing methods may not be comparable across studies, with various measurements, such as complete inhibition (7), substantial inhibition (13), 80% growth inhibition (15), 50% growth inhibition (10), or CFU quantification (9), being used. Although the antifungal activity of LF has been described for numerous yeasts and molds, most work has focused on *Candida*, due to its significance as a major human pathogen (11), and a large number of diverse strains have not been tested using the same methods and conditions, before now.

### LF is potently and consistently synergistic with AMB across a wide range of yeast species.

LF-AMB has previously been reported to be synergistic against *Cryptococcus* and *Saccharomyces* (7) and nonsynergistic against *Candida* (36), while in the current study their interaction was consistently synergistic against the majority of yeast species tested, including *Candida* (Fig. 3A; Table 2). The synergy of LF from all sources was unaffected by iron saturation. This finding is consistent with a previous report of a study with *Cryptococcus* and *Saccharomyces* that combined AMB with the iron chelators EDTA, deferoxamine, deferiprone, deferasirox, ciclopirox olamine, and LF and found that only the pairing of LF with AMB was synergistic, indicating that iron chelation alone does not enhance the effect of AMB (7). However, LF-S was less synergistic than the dairy LFs (Table 3), suggesting that, as well as influencing antifungal activity, the presence of small peptides is likely to affect the levels of synergy with AMB. This may also explain why, unlike previous studies, we found no synergistic interactions between LF and any of the other antifungal drugs tested (Fig. 3A). LF has been reported to synergize with various azole drugs and with 5-fluorocytosine in C. albicans (12–14), and synergistic interactions between azoles and the LF peptides lactoferricin and LF(1-11) have been observed (37). It is highly likely that, depending on how they are sourced and processed, different LF samples contain quite different suites of peptides. Further work is required to elucidate the peptide(s) responsible for synergy seen here with AMB.

A growing number of studies provide evidence that LF directly interacts with the fungal cell surface and acts on intracellular targets. Various mechanistic actions have been reported, including alteration of cell surface permeability and changes in membrane potential (38); mitochondrial dysfunction, causing intracellular reactive oxygen species accumulation (21, 39); and caspase activation and cytochrome *c* release (18). Similarly, the LF peptides lactoferricin and lactoferrampin are known to act by binding to and disrupting the fungal cell membrane and have been observed to be rapidly internalized by cells (20, 40). Our results support the idea of a direct fungal cell interaction, with SEM imaging showing shriveling and sunken spots in *Cryptococcus*, thinning of hyphae in C. albicans, and small pores in C. glabrata (Fig. 5C). Similar changes have been reported by others, with C. neoformans and C. albicans cells treated with LF or various peptides showing pits and collapsed cells, as well as swelling and surface blebbing (8, 9, 21).

With intracellular targets now believed to play a significant role in the antifungal activity of LF, it has been proposed that the mechanism of LF-AMB synergy may involve the increased entry of LF into the cell following disruption of the integrity of the cell wall and membrane by AMB (41). In *Cryptococcus*, substantially more cells displayed ultrastructural changes indicating cell surface damage following treatment with LF-AMB (∼40%) than following treatment with LF alone (∼10%). Interestingly, while the *Candida* species were more susceptible than the *Cryptococcus* species, very few cells or hyphae showed ultrastructural changes with any treatment (∼5%), suggesting species-specific differences in the mechanisms of synergy. Recent transcriptomic analyses of LF-AMB treatment in *Saccharomyces* and *Cryptococcus* have demonstrated species-specific differences in their response to synergistic LF-AMB treatment, with the downregulation of stress responses and the dysregulation of metal homeostasis being seen in *Saccharomyces* (41), while an increase in the stress response and disruption of transmembrane processes were seen in *Cryptococcus* (42). *Candida* species group with *Saccharomyces* in the *Saccharomycetes* class and are genetically distant from *Cryptococcus* species; hence, distinct mechanisms of synergy are likely. A range of factors could be responsible for this, including differences in external or internal targets, different metabolic and stress-response pathways, or even differences in secreted enzymes, resulting in a different suite of LF peptides being produced during incubation with the fungal cells.

### LF-AMB prevents morphological changes associated with virulence in *Candida* and *Cryptococcus*.

Many different antifungal drugs affect key virulence attributes of fungal pathogens when present at subinhibitory concentrations. For example, in *Candida* extracellular hydrolase production (43), phospholipase production (44), hemolytic activity (45), and biofilm formation (43, 46) are inhibited by various antifungal drugs, including AMB, NYS, FLC, and caspofungin. The capacity of *Candida* to make biofilms is a key virulence factor, as these protect the organism from the immune system and antifungal drug treatment, allowing it to persist inside the host (47). Here, we found that treatment with LF-AMB prevented the formation of biofilms by C. albicans and C. glabrata, reducing the amount of drug needed 4-fold for AMB and at least 128-fold for LF (Fig. 6A and B). Although it was less effective against mature biofilms, LF-AMB still reduced the amount of AMB required. In Candida albicans, the transition from yeast to hyphae also plays a role in the invasion of host tissues (48), and treatment of C. albicans with subinhibitory concentrations of LF-AMB substantially reduced the hyphal length and completely prevented hyphal formation at 1/2 FIC (Fig. 6C to E). Candida albicans has emerged as a major cause of nosocomial bloodstream infection, which is largely attributed to its capacity to form biofilms on indwelling devices (34). As biofilms and hyphal growth are difficult to treat and require higher concentrations of drug than planktonic cells (29), developing new therapies that can prevent them from forming is an area of high significance.

*Cryptococcus* cells undergo substantial morphological changes during human infection, and this can affect clinical outcomes (31). The most notable is a dramatic increase in the size of their polysaccharide capsule, and the production of giant cells (≥15 μm), micro cells (≤1 μm), irregular cells, and copious shed capsule is also seen (30, 31, 49). Previous studies have shown that subinhibitory concentrations of AMB or FLC alter capsular appearance and decrease cell size (50, 51), consistent with our results for AMB in C. deuterogattii (Fig. 6B). Although treatment with a subinhibitory concentration of LF resulted in larger capsules and only slightly inhibited the production of micro cells and shed capsule, treatment with subinhibitory concentrations LF-AMB reduced capsule size, completely blocked micro cell formation and the production of shed capsule, and increased the proportion of uninduced cells (Fig. 7C to F). Capsule is a major cryptococcal virulence factor that protects the cell from phagocytosis, downregulates the immune response, and acts as a sink for reactive oxygen species (52), while shed capsule and micro cells may enhance pathogenesis through fungal dissemination (31, 53); thus, suppression of these phenotypes has clinical relevance. In addition, the proportion of irregular cells increases with higher subinhibitory LF-AMB treatments, suggesting that these may have increased antifungal tolerance (31).

### LF-AMB has potential as a clinical antifungal treatment.

The results obtained with the G. mellonella model have been shown to correlate well with those of infection studies performed in mammalian models, and this model has been used successfully to detect *in vivo* synergistic drug treatments (54, 55). We found that while monotherapy prolonged the survival of larvae compared to that of the untreated control, LF-AMB combination therapy was significantly more effective than therapy with either LF or AMB alone (*P* < 0.0001), resulting in 83 to 93% of larvae surviving until the end of the 10-day monitoring period and reducing the fungal burden 12- to 25-fold after 24 h (Fig. 4A and B). Our work indicates that LF-AMB is effective against medically important fungi *in vivo* and could potentially be used against those causing systemic infections, like *Candida*. Various past studies have successfully trialed LF treatment in animal models and clinical tests (56). In humans, LF has been used in infant formula to protect against neonatal infection (57), and it has been tested in several clinical trials, including those exploring the use of LF for the treatment of oral candidiasis (58), Giardia lamblia infection (59), and newborn sepsis (60), with no significant adverse side effects being reported.

Although there is currently no good way to administer LF by intravenous (i.v.) injection, as its large size poses a challenge for drug delivery, new ways of i.v. administration, such as through the use of nanoparticles and dendrimers, might enable i.v. use in the future (61, 62). Dendrimers in particular have already successfully been used to intravenously administer LF for the treatment of cancer cells in mice (63). These delivery systems also bring the benefit of targeted administration at specific sites, a strategy used for liposomal AMB formulations that increases bioavailability. Peptides derived from lactoferrin are also candidates for clinical antifungal treatment and would be more amenable to i.v. administration due to their smaller size. The LF peptide LF(1-11) has been bound to bone cement to treat osteomyelitis (64) and administered via i.v. injection to treat murine infections caused by drug-resistant bacteria (65). Some LF peptides have also been used synergistically *in vivo*: lactoferricin has been coadministered with penicillin G in the treatment of mastitis in cattle (66) and with ciprofloxacin in the experimental treatment of infected mouse corneas (67). Lactoferrin and its peptides therefore make attractive adjunct therapy candidates due to their demonstrated efficacy *in vivo*, lack of side effects, potential for delivery by diverse methods, and synergistic capability.

### Conclusion.

This study has clarified some of the outstanding questions surrounding LF and its antifungal activity. We have shown that the various iron saturation levels and degrees of digestion of LF correlate with antifungal activity, suggesting a mechanistic link, and that LF is broadly active across yeast species. The synergistic pairing of LF and AMB is potent against *Candida* and *Cryptococcus*, effective in an *in vivo* model, and able to suppress known virulence mechanisms. LF-AMB thus has the potential to be developed as a novel, reliable, and effective antifungal treatment. This may be further enhanced by identifying and characterizing the LF peptides that contribute to its antifungal and synergistic activity.

## MATERIALS AND METHODS

### Fungal strains.

One hundred fungal strains were used for initial antifungal testing. These encompassed 22 yeast species (74 strains) and 24 mold species (26 strains). The majority of molds were clinical isolates from Westmead Hospital, Sydney, Australia, and the majority of yeasts from the class *Saccharomycetes* were clinical isolates from Royal North Shore Hospital, Sydney, Australia. Other strains included environmental isolates and reference strains. Yeasts from the class *Tremellomycetes* were a selection of diverse strains, including clinical, veterinary, and environmental samples from around the world. Reference strains Candida albicans SC5314, Candida glabrata CBS138, Cryptococcus neoformans H99, and Cryptococcus deuterogattii R265 were used for subsequent experiments. Full details of the strain names, sources, and countries of origin are listed in Data Set S1 in the supplemental material.

### Culture conditions.

The yeast strains were maintained as glycerol stocks at −80°C and grown on Sabouraud dextrose agar (SDA; 10 g peptone, 40 g glucose, 15 g agar, 1 liter distilled H_2_O [dH_2_O]) at 30°C for 48 h before use. Mold strains were maintained as agar cuts in water and grown on potato dextrose agar (Sigma-Aldrich) at 35°C for between 48 h and 7 days until good sporulation was obtained. Unless otherwise specified, the yeast strains were grown overnight from a single colony in 10 ml of Sabouraud dextrose broth (SDB; 10 g peptone, 20 g glucose, 1 liter dH_2_O) in a 100-ml Schott bottle at 37°C with shaking at 180 rpm until the culture reached exponential growth phase. Cells were collected by centrifugation, washed twice with PBS, and counted with a hemocytometer. All assays were performed with technical duplicates, and at least three biological replicates were performed on separate days. The final inoculum concentration was confirmed by backplating.

### LF and antifungal drugs.

Three sources of bovine lactoferrin (LF) were used in this study: one purchased from Sigma-Aldrich (LF-S); one referred to as LF from dairy 1 (LF-D1), supplied by Bega Bionutrients; and one referred to as LF from dairy 2 (LF-D2), supplied by Fonterra. The antifungal drugs used included amphotericin B (AMB), nystatin (NYS), fluconazole (FLC), itraconazole (ITC), voriconazole (VRC), and 5-fluorocytosine (5FC). AMB, NYS, VRC, and 5FC were purchased from Sigma-Aldrich; FLC and ITC were purchased from Sapphire Bioscience. Stock solutions were prepared following CLSI guidelines for antifungal susceptibility (68). These were stored at −80°C and used within 6 months.

### Preparation of holo- and apo-LF.

Holo- and apo-forms of LF were prepared based on previously published methods, with slight modifications (69–71). For holo-lactoferrin (holo-LF), 20 mg/ml of each LF sample was dissolved in 100 mM citrate phosphate buffer (pH 7.4) and mixed with freshly prepared ferric nitriloacetic acid solution (FeNTA; pH 7.4) containing 9.9 mM ferric nitrate and 8.5 mM nitriloacetic acid, a weak chelating agent, to achieve an LF/iron molar ratio of 1:2. The mixture was incubated at ambient temperature for 1 h to allow LF to fully saturate with iron, and then the excess iron was removed by dialysis against Milli-Q water using a cellulose membrane for 48 h with constant stirring and four changes of water. For apo-lactoferrin (apo-LF), 20 mg/ml of each LF sample was dissolved in ultrapure water and was dialyzed extensively, using a cellulose membrane, against 100 mM citrate buffer (0.9 mM sodium citrate dihydrate, 9.1 mM citric acid, pH 3.0) for 24 h with constant stirring and two changes of buffer to remove ferric ions and then against Milli-Q water for a further 24 h with constant stirring.

### LF characterization by SDS-PAGE.

LF samples dissolved in Milli-Q water at various concentrations were analyzed by SDS-PAGE on 10 to 20% Criterion TGX precast midi protein gels (Bio-Rad) with Tris-Tricine running buffer (100 mM Tris, 100 mM Tricine, 0.1% SDS). Samples were diluted 1:1 with Laemmli sample buffer (Bio-Rad) and β-mercaptoethanol, heated at 90°C for 5 min, and briefly centrifuged prior to being loaded onto the gels. A 20-μl aliquot of each sample was loaded alongside 2 μl of a broad-range unstained protein standard (New England Biolabs), and the gels were run at 200 V for 45 min. The gels were washed three times for 5 min each time in 200 ml of Milli-Q water to remove SDS and drained, before being covered with 50 ml Bio-Safe Coomassie stain (Bio-Rad) and incubated at room temperature with gentle shaking for 1 h. The gels were then washed once again in 200 ml of Milli-Q water for 30 min and photographed using a digital camera.

### LF characterization by mass spectrometry.

Matrix-assisted laser desorption ionization–time of flight (MALDI-TOF) mass spectrometry (MS) was used to analyze the composition of the LF samples. A 1-μl aliquot of matrix solution A (sinapinic acid [SA] saturated in ethanol [EtOH]) was applied to the ground steel target in a thin layer. Samples were then mixed 1:1 with matrix solution B (SA saturated in 0.1% trifluoroacetic acid, 30% acetonitrile [TA30]), and 0.5 μl was applied on top of the matrix solution A layer and allowed to dry. Mass spectra were acquired in linear mode using a laser power of 85% from the sum of 10,000 laser shots. MALDI-TOF mass spectra were baseline corrected (precision, 75; relative offset, 25) and smoothed (Savitzky-Golay, 5-*m/z* window, 5 cycles), and figures were generated using the mMass program (72). Inductively coupled plasma mass spectrometry (ICP-MS) was used to measure the iron concentration in the original LF sample and the prepared apo-LF and holo-LF samples. Samples were diluted to 100 μg/ml in 500 μl ultrapure water (Sigma) and digested with 100 μl of concentrated nitric acid overnight. Samples were further diluted to a range of concentrations and run on a PerkinElmer NexION 300 ICP-MS using the ^57^Fe isotope for quantification of the iron content.

### Antifungal susceptibility testing by broth microdilution.

Antifungal susceptibility testing was performed by the broth microdilution methodology in 96-well microtiter plates in accordance with CLSI guidelines for yeasts and filamentous fungi (68, 73). Fungal inocula were prepared from colonies growing on agar plates to a final concentration of 0.5 × 10^3^ to 2.5 × 10^3^ CFU/ml for yeasts, 0.4 × 10^4^ to 5 × 10^4^ CFU/ml for nondermatophyte molds, and 1 × 10^3^ to 3 × 10^3^ CFU/ml for dermatophytes. All tests used RPMI 1640 (Sigma-Aldrich) supplemented with 0.165 M MOPS (morpholinepropanesulfonic acid) and 2% d-glucose, except for *Cryptococcus* spp., for which yeast nitrogen base (Sigma-Aldrich) supplemented with 0.165 M MOPS and 0.5% d-glucose was used. Drugs were assayed at concentration ranges of 0.0039 to 4 μg/ml for AMB, NYS, ITC, and VRC; 0.0625 to 64 μg/ml for FLC and 5FC; and 1 to 1,024 μg/ml for LF. For MICs, plates were incubated without agitation at 35°C for 24 h (*Mucorales*), 48 h (*Dothideomycetes*, *Saccharomycetes*, *Aspergillus* spp., *Fusarium* spp.), 72 h (*Cryptococcus* spp., *Lomentospora* spp., *Scedosporium* spp.), or 96 h (dermatophytes). The MIC was determined visually and was defined as the lowest drug concentration at which growth was inhibited 100% for LF (yeasts, nondermatophyte molds, and dermatophytes), 100% for AMB and NYS (yeasts), and 50% for FLC, ITC, VRC, and 5FC. For *Candida* species only, the MICs for AMB, FLC, ITC, VRC, and 5FC were obtained from clinical lab tests at Royal North Shore Hospital using a Sensititre YeastOne YO10 antifungal susceptibility testing plate (Thermo Scientific). The plate was prepared and the results were interpreted according to the manufacturer’s instructions and CLSI guidelines, as described above. MIC data were plotted visually using the iTOL (version 4.4.2) program (74). For time-kill curves, the plates were incubated at 35°C with shaking at 180 rpm in a Tecan Spark microplate reader, and cell density was measured every 2 h by determination of the absorbance at 600 nm for a total of 48 h. Each test plate included the reference strain Candida parapsilosis ATCC 22019.

### Drug interaction testing by checkerboard assay.

Checkerboard assays were used to determine pairwise interactions between LF and the other antifungal drugs against the four yeast reference strains (75). Serial 2-fold dilutions starting at 4× MIC of LF and each interacting drug were prepared and plated in 96-well microtiter plates in the horizontal and vertical directions, respectively. Inoculum preparation, the media, and the incubation conditions were those described above for antifungal susceptibility testing. Checkerboard assay results were assessed both by visual examination and by determination of the absorbance at 600 nm (BioTek ELx800 absorbance reader). An initial determination of the drug interactions against 22 yeast species used an abbreviated diagonal-sampling checkerboard method (23).

### Models used for assessment of drug interactions.

Two models were used to assess drug interactions. The fractional inhibitory concentration index (FICI), which is based on the Loewe additivity model, determines the fractional inhibitory concentration (FIC) of each drug in the pair as (MIC*_X_*/MIC*_Y_*), where MIC*_X_* is the MIC of the drug alone and MIC*_Y_* is the MIC of the drug in combination. FICI is then calculated as FIC_drug A_ + FIC_drug B_. This model defines interactions as synergistic (≤0.5), indifferent (>0.5 to 4), or antagonistic (>4) (76). The MacSynergy II program is based on the Bliss independent model and uses the equation *E*_AB_ = *E*_A_ + *E*_B_ − (*E*_A_*E*_B_) where *E*_AB_ is the additive effect of drugs A and B predicted by their individual effects (*E_A_* and *E_B_*) (77). MacSynergy II generates a three-dimensional interaction surface by calculating the predicted indifferent effect and representing this as a flat plane, with peaks and troughs representing synergistic and antagonistic interactions, respectively. This model uses interaction volumes (micromolar squared percent) and defines positive volumes as synergistic and negative volumes as antagonistic. It additionally defines interactions within these categories as insignificant (≤25 μM^2^%), minor (>25 to 50 μM^2^%), moderate (>50 to 100 μM^2^%), or strong (<100 μM^2^%).

### Galleria mellonella infection assays.

The Galleria mellonella larvae used in this study were obtained following oviposition of adult wax moths. The larvae were maintained at 26°C and reared on an artificial diet (250 g fine multigrain cereal, 58.3 g organic Australian honey, 58.3 g glycerol, 10 g distilled water, 8 g dried baker’s yeast) until they reached the final larval instar stage. For each test group, 10 larvae with similar sizes (2 to 3 cm) and no marks or discoloration were placed in petri dishes and starved for 24 h. An inoculum of 10^8^ cells was prepared by resuspension of cells in phosphate-buffered saline (PBS; Oxoid). The injection area was cleaned using an ethanol swab, and 10 μl of undiluted inoculum was injected through the last left proleg of each larva into the hemolymph using a 50-μl Hamilton syringe. After 30 min, the antifungal agents were injected using the same technique. All antifungal agents were administered once by separate injections, with a different proleg being used for each injection. Each test group was standardized with the same number and volume of injections, with PBS being used in place of inoculum or drug, as appropriate. A group of larvae injected only with PBS was included to monitor the effects of physical injury from the injection, and a group of untouched larvae was included as an untreated control. After injection, each test group of larvae was placed into a clean petri dish and incubated at 35°C. The survival or death of each larva was recorded at 24-h intervals over a period of 10 days. For fungal burden quantification, three randomly chosen larvae were washed with ethanol, cut into small pieces with a scalpel, and homogenized in an Eppendorf tube. The homogenate was serially diluted twice, and 50 μl of the resulting dilutions was inoculated onto SDA plates. The plates were incubated at 35°C for 48 h before colonies were enumerated. Three independent biological replicates were performed on different days, and the inoculum concentration was confirmed by backplating.

### Scanning electron microscopy.

Coverslips were prepared by rinsing with 90% acetone followed by Milli-Q water, incubation in a 1% polyethyleneimine (PEI) solution for 60 min, rinsing with Milli-Q water twice, and air drying at 60°C for 10 min. PEI-coated coverslips were then placed into the wells of a 12-well tissue culture plate, enough sample was added to completely submerge the coverslips, and the cells were allowed to settle for 60 min. The coverslips were then washed with 0.1 M phosphate buffer (PB), 1 ml of fixative (2.5% glutaraldehyde, 2% paraformaldehyde, 0.1 M PB) was added, and these were left overnight at 4°C. The coverslips were next washed three times in 0.1 M PB, secondary fixative (1% osmium tetroxide, 0.1 M PB) was added, and the samples were incubated for 2 h. Coverslips were rinsed in Milli-Q water three times for 5 min each time before the cells were dehydrated in a series of graded ethanol solutions (30, 50, and 70% EtOH twice for 5 min each time, 95 and 100% EtOH three times for 5 min each time). Cells were chemically dried by adding 100% hexamethyldisilazane (HMDS) for 2 min. The HDMS was then removed by aspiration, and any remaining HMDS was evaporated in a fume hood. Coverslips were mounted onto metallic stubs, sputter coated with gold for 2 min at 2 mA in an Emitech K550X sputter coater, and visualized in a JEOL JCM-6000 scanning electron microscope operating at 15 kV.

### *Candida* biofilm assays.

*Candida* biofilm assays were performed based on previously published methods with slight modifications (78). *Candida* cultures were diluted to a concentration of 10^6^ cells/ml in DMEM, and 100 μl was pipetted into individual wells of a 96-well microtiter plate. The plates were sealed with Parafilm and incubated at 37°C for either 4 h (initial adherence) or 24 h (complex three-dimensional architecture). The medium was then carefully aspirated from the wells, taking care not to disrupt the biofilms, and the wells were washed twice with 200 μl of PBS to remove any planktonic or nonadherent cells. The plates were drained in an inverted position for 5 min to remove any residual PBS, and antifungal agents were added to the wells in 200 μl of medium. The plates were again sealed with Parafilm and incubated at 37°C for 24 h. Once this time had passed, 100 μl of XTT-menadione solution (0.5 g/liter XTT in PBS, 1 μM menadione in acetone) was added to each well and the plates were covered in aluminum foil and incubated in the dark at 37°C for 4 h. Mitochondrial dehydrogenases in live cells reduce the XTT tetrazolium salt to XTT formazan, resulting in a colorimetric change in the supernatant, which was assayed by transferring 80 μl into new wells and measuring the absorbance at 500 nm (BioTek ELx800 absorbance reader).

### *Candida* hyphal induction assays.

*Candida* cultures were diluted to a concentration of 10^6^ cells/ml in DMEM. From this, 10 ml was pipetted into 100-ml Schott bottles, and these either were left untreated or were treated with individual drugs at 1/2, 1/4, and 1/8 MIC or 1/2, 1/4, and 1/8 FIC. All cultures were incubated at 37°C with shaking at 180 rpm. At 3, 6, and 12 h, 500-μl aliquots from each treatment were taken, wet mounts were prepared, and slides were photographed using an IS10000 inverted microscope under a 40× objective and ISCapture imaging software (Tucsen Photonics). Hyphal length was measured for 100 cells per treatment using ImageJ software (National Institutes of Health).

### *Cryptococcus* capsule induction assays.

*Cryptococcus* capsule induction assays were performed based on previously published methods, with slight modifications (31). *Cryptococcus* cultures were diluted to a concentration of 2 × 10^4^ cells/ml in Dulbecco’s modified Eagle medium (DMEM; Life Technologies); 5-ml aliquots were then pipetted into individual wells of a 6-well tissue culture plate, and these were either left untreated or were treated with individual drugs at 1/2, 1/4, or 1/8 MIC or at 1/2, 1/4, or 1/8 FIC. The plates were sealed with Parafilm and incubated with 5% CO_2_ at 37°C for 5 days. To visualize capsule, 1 ml of culture was resuspended in 150 μl of PBS and counterstained with 20 μl of India ink. A 15-μl aliquot of this mixture was placed on a glass slide and dried for 10 min under a coverslip. The slides were then photographed using an IS10000 inverted microscope (Luminoptic) under a 40× objective and ISCapture imaging software (Tucsen Photonics). The total diameter (including capsule) (*d_t_*) and the yeast cell diameter (*d_y_*) were measured for 100 cells per treatment using ImageJ software (National Institutes of Health). From these measurements, capsule thickness (*t_c_*) was calculated as 1/2(*d_t_* – *d_y_*). Total volume (*v_t_*) and yeast cell volume (*v_y_*) were calculated using the formula for the volume of a sphere [4/3(π*r_d_*^3^)]. Cells with a *d_y_* of less than 1 μm were identified as micro cells and were noted along with shed capsule and morphologically irregular cells.

### Statistical analysis.

Significant differences between treatment groups for the *Galleria* infection assays were determined using the log-rank test to compare the distributions of two samples. Significant differences between treatment groups for capsule and hyphal measurements were determined using two-tailed unpaired *t* tests with Welch’s correction, and differences in variance were assessed by F test analysis. *P* values of <0.05 were considered significant. Error bars represent the mean ± 95% confidence intervals. Data were analyzed using Excel (Microsoft Corporation) and Prism (version 5) software (GraphPad Inc.).

## Supplementary Material

Supplemental file 1

Supplemental file 2

Supplemental file 3
